# Asymptomatic Pneumococcal Carriage, Antimicrobial Resistance, and Associated Risk Factors Among Paediatric Healthcare Workers in Benin

**DOI:** 10.3390/tropicalmed10090263

**Published:** 2025-09-15

**Authors:** Chakir Ishola Bello, Cyriaque Comlan Degbey, Yves Eric Denon, Adolphe Adjanonhoun, Lamine Baba-Moussa

**Affiliations:** 1 Laboratory of Biology and Molecular Typing in Microbiology, Faculty of Sciences and Techniques, University of Abomey-Calavi, Cotonou P.O. Box 1604, Benin; laminesaid@yahoo.fr; 2 Department of Environmental Health, Regional Institute of Public Health, University of Abomey-Calavi, Ouidah P.O. Box 384, Benin; comlancy@yahoo.fr; 3 University Hospital Hygiene Clinic, Hubert Koutoukou Maga National Teaching Hospital (CNHU-HKM), Cotonou P.O. Box 386, Benin; 4 Diagnostic and Exploratory Department, Ministry of Health of Benin, Cotonou P.O. Box 882, Benin; denric2000@gmail.com; 5 National Institute of Agricultural Research of Benin (INRAB), Cotonou P.O. Box 884, Benin; adjanohouna@yahoo.fr

**Keywords:** oropharyngeal, nasopharyngeal, healthcare professional, asymptomatic carriage, paediatric units, multidrug resistance, *Streptococcus pneumoniae*

## Abstract

Healthcare professionals (HCPs) working in paediatric settings are routinely exposed to respiratory pathogens, increasing their risk of asymptomatic colonisation by meningitis-associated bacteria. This study is the first to assess oropharyngeal and nasopharyngeal carriage of major bacterial meningitis pathogens among paediatric HCPs in Benin, and to identify associated risk factors. A cross-sectional analytical study was conducted in nine hospitals between 1 September 2023 and 30 September 2024. Data collection involved a structured questionnaire and paired oropharyngeal and nasopharyngeal swabs. Culture-based identification and antimicrobial susceptibility testing were performed according to CA-SFM guidelines. By culture method, *Streptococcus pneumoniae* was the most frequently isolated pathogen, mainly from oropharyngeal samples (47.5%). Most of these strains exhibited multidrug resistance. In nasopharyngeal samples analysed by real-time PCR, detection rates for *S. pneumoniae* were markedly higher (24.4%) compared to culture (5.0%), highlighting the limited sensitivity of conventional methods in detecting asymptomatic carriage. Pneumococcal colonisation was significantly associated with recent respiratory tract infections, and residence in high-risk areas (*p* < 0.05). These findings underscore the need for enhanced molecular surveillance, along with strengthened infection control measures and targeted vaccination strategies, to mitigate the risk of horizontal transmission in paediatric wards.

## 1. Introduction

Meningitis is a severe and potentially fatal infection of the meninges, the membranes that envelop the brain and spinal cord. Despite the implementation of conjugate vaccines targeting key pneumococcal and meningococcal serotypes through routine childhood immunisation and mass vaccination campaigns, the global burden of meningitis remains considerable. This is particularly true in the African meningitis belt, from Senegal in the west to Ethiopia in the east, including 26 countries, where outbreaks continue to occur due to the circulation of non-vaccine meningococcal serogroups and a persistently high incidence of pneumococcal meningitis [1]. Although a range of pathogens can cause meningitis, *Streptococcus pneumoniae*, *Neisseria meningitidis*, and *Haemophilus influenzae type b* are the most frequently implicated worldwide. Incidence rates in high-income countries, such as the United States and those in Europe, range between 0.7 and 0.9 per 100,000 population annually [2,3]. In contrast, studies from Africa report much higher rates, up to 40 per 100,000 population [2,4]. In Benin, a country located within the meningitis belt, the median age of confirmed cases is four years, and the incidence was reported at 3.2 per 100,000 inhabitants in 2018, following a peak of 6.3 in 2016 [5]. Transmission of meningitis-causing bacteria occurs through respiratory droplets, with close or prolonged contact facilitating spread. Colonisation of the nasopharynx by these pathogens is typically asymptomatic but is a necessary precursor to invasive disease. Carriage rates are highest among infants and young children, with prevalence exceeding 60% in certain settings [6]. While the age-related dynamics of bacterial carriage are well described in Europe and North America, there is a paucity of data from countries within the meningitis belt. 

Healthcare professionals (HCPs) working in paediatric settings are at increased risk of exposure to respiratory pathogens due to their frequent contact with symptomatic children. In Benin, acute respiratory infections are the second most common reason for medical consultation among children under five, accounting for 17% of cases after malaria (48.8%) [7]. This environment fosters the horizontal transmission of encapsulated bacteria such as *S. pneumoniae*, *N. meningitidis*, and *H. influenzae*, which may lead to increased asymptomatic colonisation among frontline staff. Given that conventional culture methods have a lower sensitivity compared to PCR for detecting asymptomatic carriage in adults, and that detection rates may differ between oropharyngeal and nasopharyngeal sites, we employed both sampling sites and complementary methods to obtain a more accurate estimate of pneumococcal carriage and to facilitate reliable interpretation of the results.

Despite the potential risk, little is known about the prevalence and determinants of nasopharyngeal or oropharyngeal carriage of these pathogens among healthcare workers in paediatric units in Benin or across West Africa. Understanding colonisation patterns in this population is essential for evaluating nosocomial transmission risks and informing infection control strategies.

This study aimed to assess the prevalence of asymptomatic carriage of *S. pneumoniae* and its antimicrobial resistance profile among healthcare professionals in paediatric departments in Benin as well as to identify associated risk factors.

## 2. Materials and Methods

We conducted a cross-sectional analytical study to assess the prevalence of asymptomatic carriage of meningitis-associated bacteria among healthcare professionals working in paediatric units, and to identify potential risk factors. This section outlines the study setting, participant selection criteria, procedures for sample collection and laboratory analysis, antimicrobial susceptibility testing, and the statistical methods used for data analysis.

### 2.1. Study Design

The study was designed as a cross-sectional investigation conducted between 1 September 2023 and 30 September 2024 in nine public hospitals across northern and southern Benin. The selection of the facilities was based on the presence of operational paediatric and neonatology units. The same healthcare facilities included in our previous study [8] were selected for this investigation, and a detailed map of the sites is available in that publication.

In accordance with WHO guidelines on standard precautions, all included hospitals were expected to implement infection prevention measures, including the routine use of personal protective equipment.

### 2.2. Study Population and Setting

Benin is a West African country with an area of 114,763 square kilometres and a population of approximately 13.71 million. It shares borders with Burkina Faso and Niger to the north, Togo to the west, Nigeria to the east, and the Atlantic Ocean to the south. The climate varies from equatorial in the south to tropical in the centre and semi-arid in the far north. The northern region of the country is most frequently affected by bacterial meningitis outbreaks. This study targeted paediatric healthcare professionals (HCPs) working in hospitals located in both epidemic-prone and non-epidemic regions. In the northern part of the country, two hospitals from the Borgou Department and two from the Atacora Department were included. In the southern region, the study covered the Cotonou Reference Hospital, the Mother and Child University Hospital, the Suru Lere University Hospital, the Mono-Couffo Departmental Hospital Centre, and the Lokossa Zonal Hospital [8]. 

All participating hospitals regularly receive suspected cases of meningitis. The target population consisted of paediatricians, general practitioners, nurses, and nursing assistants who were actively working in paediatric units during the study period.

### 2.3. Inclusion and Exclusion Criteria

#### 2.3.1. Inclusion Criteria

Healthcare professionals were eligible for inclusion if they were present in the targeted paediatric or neonatology departments at the time of the survey, were in direct contact with children or neonates, and provided informed consent for participation and sample collection.

#### 2.3.2. Exclusion Criteria

Administrative staff and personnel not directly involved in patient care were excluded from the study. In addition, healthcare workers who declined participation or were unable to undergo sample collection for medical reasons, such as active nasal bleeding or ongoing antibiotic treatment, were also excluded.

### 2.4. Sample Size Calculation

The sample size was estimated using Cochran’s formula: *n* = (*Z*^2^*α* × *P* × *Q*)/i^2^, where: P is the estimated prevalence, Q = 1 − P, Zα is the Z-value corresponding to the desired confidence level (1.96 for 95%), and i is the desired precision.

Based on previous data from the African meningitis belt, specifically Burkina Faso, the prevalence of nasopharyngeal carriage of *Streptococcus pneumoniae* among adults was estimated at 22% [9]. Thus, P = 0.22, Q = 0.78, Zα = 1.96, and i = 0.06.

The minimum calculated sample size was 183 participants. To account for potential dropouts, refusals, or unusable data, a 20% adjustment was applied, increasing the target sample size to approximately 220 participants. Ultimately, a total of 261 healthcare professionals were consecutively recruited, thereby enhancing the statistical power and precision of the study estimates.

### 2.5. Bacterial Sampling and Identification

Sampling and detection procedures followed the standardised guidelines of the WHO Pneumococcal Carriage Working Group [10]. For each paediatric healthcare professional, one oropharyngeal and one nasopharyngeal swab were obtained. All participants were asymptomatic and exhibited no signs of active infection at the time of sampling. Swabs were collected using sterile equipment, kept at a cool temperature, and inoculated onto culture media within two hours.

Bacterial culture was performed on selective chocolate agar and blood agar plates (Oxoid, Thermo Fisher Scientific, Basingstoke, UK); the latter were supplemented with 5% defibrinated sheep blood and antibiotics, nalidixic acid and colistin (NAC), to enhance selectivity. Plates were incubated at 37 °C in a 5% CO_2_ atmosphere for 24 h. Positive cultures were examined using standard microbiological techniques, including colony morphology, Gram staining, and biochemical assays such as oxidase, catalase, motility assays, and the optochin susceptibility test for alpha-haemolytic colonies suggestive of *Streptococcus pneumoniae*.

Due to limited reagent availability, molecular analysis by real-time quantitative polymerase chain reaction (qPCR) was performed only on nasopharyngeal samples. DNA detection was conducted using the AriaMx Real-Time PCR System (Agilent Technologies, Santa Clara, CA, USA), employing TaqMan probe-based fluorescence technology. This protocol enabled specific and sensitive detection of target pathogen DNA. The following gene targets were used: lytA for *Streptococcus pneumoniae*, sodC for *Neisseria meningitidis*, and hpd3 for *Haemophilus influenzae*. Fluorescence was recorded at the end of each amplification cycle, and cycle threshold (Ct) values were automatically generated by the AriaMx software, version 2.1. Samples were considered positive for *S. pneumoniae*, *N. meningitidis*, and *Haemophilus influenzae* when the specific gene target yielded a cycle threshold (Ct) value of ≤ 35. These molecular data, obtained from the same participants as in the present study, have been previously published [8] and are presented here solely for comparison with culture-based detection to assess concordance and site-specific sensitivity.

### 2.6. Antimicrobial Susceptibility Testing

The antibiotic susceptibility of all *Streptococcus pneumoniae* isolates was assessed using the Kirby–Bauer disc diffusion method, in accordance with the 2024 guidelines of the Antibiogram Committee of the French Society for Microbiology (CA-SFM/EUCAST). The antibiotics tested included oxacillin, levofloxacin, cotrimoxazole, erythromycin, clindamycin, tetracycline, chloramphenicol, and vancomycin. Antibiotic discs were sourced from Oxoid (Thermo Fisher Scientific, Basingstoke, UK). For *Streptococcus pneumoniae*, the oxacillin (1 µg) disc was used as a surrogate marker to predict susceptibility to penicillin and other β-lactams, in line with guideline recommendations. Oxacillin is not used in routine clinical management of pneumococcal infections. In this study, it was included solely for screening and epidemiological purposes. Quality control strains (ATCC 49619 for *S. pneumoniae*) were used to ensure accuracy.

### 2.7. Data Collection and Variables

All participants received detailed information about the study and provided written informed consent prior to enrolment. Each individual then completed a structured, standardised questionnaire developed specifically for this study (see Supplementary File S1). The questionnaire was pilot-tested to ensure clarity, relevance, and comprehensibility prior to implementation.

The instrument collected data on socio-demographic characteristics (age, sex, and professional category), occupational exposure (hospital unit), and vaccination status against *Streptococcus pneumoniae*, *Neisseria meningitidis*, and *Haemophilus influenzae type b*, as well as recent history of respiratory symptoms and household cohabitation with children under 10 years of age.

The primary outcome variable was asymptomatic colonisation with *S. pneumoniae*, *N. meningitidis*, or *H. influenzae*, confirmed by culture. For comparison with culture results, previously published real-time PCR data from the same participants, nasopharyngeal swabs were used (221 of 261 samples analysed; Ct ≤ 35 considered positive). Due to reagent limitations, PCR was performed only on nasopharyngeal samples, which constitutes a predefined limitation of this comparison. Independent variables included age group, professional category, geographical region of residence, vaccination status, recent respiratory tract infections, and contact with young children.

All variables were categorised for analysis. Age was grouped into four categories: 18-24 years, 25-34 years, 35-44 years, and over 45 years, with the upper bound of each category inclusive. Colonisation was considered present when at least one of the target bacteria was detected in either the oropharyngeal or nasopharyngeal swab.

### 2.8. Statistical Analysis 

Data collected during the study were coded and analysed using IBM SPSS Statistics version 30.0 (IBM Corp., Armonk, NY, USA), with daily data entry performed in Microsoft Excel 2019 (Microsoft Corporation, Redmond, WA, USA). Categorical variables were summarised as frequencies and percentages. Associations between independent categorical variables and asymptomatic colonisation by the primary epidemic-causing meningitis pathogens were assessed using the Pearson chi-square test. A *p*-value < 0.05 was considered statistically significant. In addition, the sensitivity of culture compared with real-time PCR for detecting asymptomatic carriage was evaluated descriptively, with proportions of positive results presented side by side. Variables with *p*-values < 0.2 in bivariate analysis were included in multivariate logistic regression models to identify independent predictors of carriage. Odds ratios (ORs) with 95% CIs were reported, and statistical significance was set at *p* < 0.05.

## 3. Results

### 3.1. Characteristics of Healthcare Workers

A total of 261 healthcare professionals participated in the study and completed the questionnaire in full. The sociodemographic and occupational characteristics of the participants are summarised in Table 1.

The majority of respondents were young adults aged 18 to 35 years (*n* = 173; 66.2%) and female (*n* = 159; 60.9%). Nurses constituted the largest professional category (*n* = 127; 48.7%), followed by general practitioners and nursing assistants. Most participants were assigned to paediatric units (*n* = 135; 51.7%). Geographically, the highest proportion of healthcare workers was recruited from the Littoral region (*n* = 113; 43.3%). The highest number of participants was recruited from the Littoral region, as previously illustrated in our prior study [9].

### 3.2. Asymptomatic Carriage Prevalence

A total of 261 nasopharyngeal and 261 oropharyngeal swab samples were collected from participating healthcare workers. Classical culture-based methods enabled the identification of *Streptococcus pneumoniae* and *Haemophilus influenzae* from both sampling sites.

Overall, oropharyngeal samples yielded a higher carriage rate, with *S. pneumoniae* detected in 124/261 (47.5%) oropharyngeal samples versus 13/261 (5.0%) nasopharyngeal samples. No isolates of *Neisseria meningitidis* were detected in any of the samples (Table 2).

### 3.3. Comparison Between Culture and Real-Time PCR Detection

The comparison between conventional culture results and those obtained through real-time PCR clearly demonstrated a marked difference in sensitivity between the two methods in nasopharyngeal samples. While culture-based techniques identified *Streptococcus pneumoniae* in only 5.0% (13/261) of participants and *Haemophilus influenzae* in 0.38% (1/261), no *Neisseria meningitidis* isolates were recovered by culture.

In contrast, molecular detection using real-time PCR revealed significantly higher rates of asymptomatic carriage. The lytA gene of *S. pneumoniae* was detected in 24.4% (54/221) of nasopharyngeal samples, the hpd3 gene of *H. influenzae* in 11.8% (26/221), and the sodC gene of *N. meningitidis* in 10.0% (22/221).

**Figure 1:**
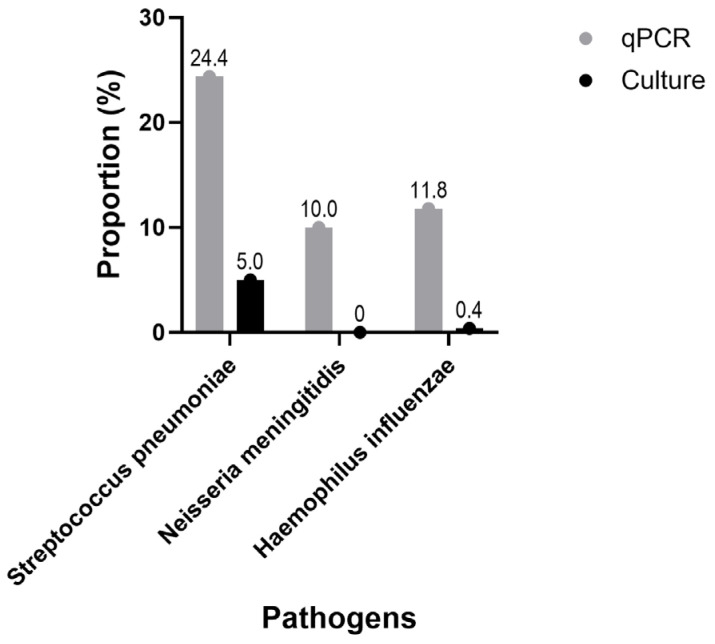
Comparison of nasopharyngeal pathogen carriage prevalence according to the detection method used (culture vs. real-time PCR).

These findings highlight the limited sensitivity of culture, particularly for asymptomatic carriers, and underscore the added value of molecular tools for more accurate and reliable detection of meningitis-causing pathogens, especially in the context of hospital-based epidemiological surveillance (see Figure 1).

### 3.4. Antimicrobial Susceptibility Profile of Streptococcus pneumoniae Isolates

The antibiotic susceptibility of all *Streptococcus pneumoniae* isolates was assessed and interpreted in accordance with the 2024 guidelines of the Antibiotic Susceptibility Committee of the French Society for Microbiology (CA-SFM 2024). All isolates were resistant to cotrimoxazole (100%). The majority exhibited decreased susceptibility to penicillin (97%) and high levels of resistance to tetracycline (95%). Only 21% of isolates remained susceptible to levofloxacin.

**Figure 2:**
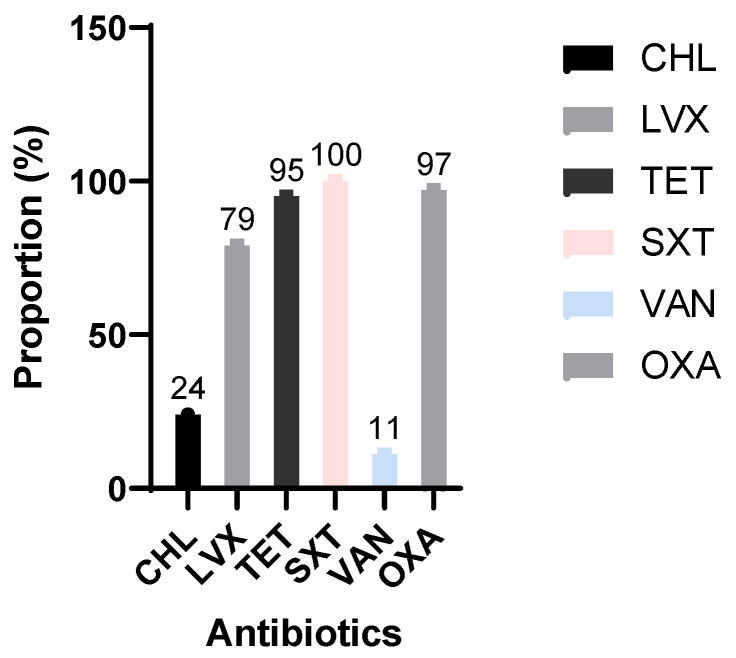
Resistance patterns of *Streptococcus pneumoniae* isolates as determined by the Kirby–Bauer disc diffusion method, interpreted according to CA-SFM 2024 guidelines. CHL = chloramphenicol; LVX = levofloxacin; TET = tetracycline; SXT = cotrimoxazole; OXA = oxacillin; VAN = vancomycin.

By contrast, a greater proportion of strains were susceptible to chloramphenicol (76%) and vancomycin (89%) (Figure 2).

### 3.5. Sociodemographic Factors Associated with Pneumococcal Colonisation in Paediatric Healthcare Workers

The analysis of risk factors was conducted based exclusively on culture-positive cases of *Streptococcus pneumoniae* among asymptomatic healthcare workers. This approach aimed to explore sociodemographic associations that may inform targeted prevention efforts in paediatric settings (Table 3).

Among the 261 participants, pneumococcal colonisation was significantly more common among healthcare workers in paediatric wards (63.7%) compared to those in neonatology units (32.0%, *p* < 0.001). Although some *p*-values were below 0.05 (age group *p* = 0.021; profession *p* = 0.001), the corresponding odds ratios all included 1 (sex OR 0.79 [0.4–1.2]; age OR 1.02 [0.6–1.4]; profession OR 1.31 [0.8–1.7]), indicating that these associations were not statistically significant when accounting for effect size. No significant association was found between sociodemographic factors and colonisation.

### 3.6. Factors Associated with Pneumococcal Carriage Among Healthcare Workers

The study identified several factors significantly associated with colonisation by the major meningitis-causing pathogen *Streptococcus pneumoniae* (Table 4). Recent respiratory tract infection within the past two weeks (OR 1.3 [1.1–3.4], *p* = 0.022), vaccination status (OR 1.1 [0.4–1.6], *p* = 0.002), and residence in the meningitis belt of northern Benin (OR 1.9 [1.3–4.1], *p* = 0.001) were associated with elevated colonisation rates. However, as the odds ratio for vaccination status included 1, this association was not statistically significant when considering effect size. Although mask usage during patient care was associated with reduced colonisation, this reduction did not reach statistical significance. Cohabitation with children under 10 years also showed no significant association (OR 0.79 [0.7–2.2], *p* = 0.091).

## 4. Discussion

Meningitis continues to pose a major public health threat, particularly in paediatric populations, where asymptomatic colonisation by bacterial pathogens often precedes invasive disease. *Streptococcus pneumoniae*, *Neisseria meningitidis*, and *Haemophilus influenzae* are commensal organisms of the human upper respiratory tract, acting as reservoirs for horizontal transmission and potential progression to disease [11]. This study examined the prevalence, risk factors, and antimicrobial resistance profile of *Streptococcus pneumoniae* among healthy paediatric healthcare workers in Benin.

Our results revealed low detection rate of *Streptococcus pneumoniae* in nasopharyngeal samples (5%) by culture, and higher detection in oropharyngeal samples (47.5%), while *Neisseria meningitidis* was not isolated. In contrast, molecular detection by real-time PCR, performed exclusively on nasopharyngeal samples, revealed substantially higher carriage rates: 24.4% for *S. pneumoniae*, 11.8% for *H. influenzae*, and 10.0% for *N. meningitidis* [8]. These findings underscore the well-documented limitations of classical culture methods, particularly in detecting low-density colonisation in asymptomatic individuals, and highlight the superior sensitivity of molecular techniques for epidemiological surveillance [12].

Our findings are consistent with those of Steurer et al., who reported low nasopharyngeal colonisation rates using culture-based methods, 0.45% in nasopharyngeal and 4.8% in oropharyngeal swabs, among a similar cohort of healthcare professionals [12]. In contrast, Subramanya et al. reported much higher oropharyngeal colonisation rates (65%) with *S. pneumoniae* among Indian healthcare workers, while Amritha et al. reported a 30% overall carriage for *S. pneumoniae* and *H. influenzae* in a comparable setting [13,14]. Notably, our study demonstrated a significant difference between nasopharyngeal and oropharyngeal detection rates, likely reflecting both anatomical and methodological influences as well as region-specific differences in bacterial circulation and vaccine uptake.

While younger healthcare workers, particularly those aged 18–34 years, and general practitioners showed elevated colonisation rates, however, the odds ratio included 1, indicating that these associations were not statistically significant when considering effect size. Similarly, differences in colonisation according to work setting (paediatrics vs. neonatology) and sex were not statistically significant. These findings suggest that, in our study population, demographic and professional factors were not clearly associated with pneumococcal carriage. Previous studies have reported mixed results: Samadpanah et al. observed higher carriage rates among nurses [15], whereas Waghela et al. in Connecticut, USA, found no significant associations between demographic characteristics and pneumococcal detection [16]. Such discrepancies likely reflect differences in clinical duties, patient contact intensity, healthcare system organisation, and regional variations in occupational exposure.

The antimicrobial susceptibility profile of *S. pneumoniae* isolates revealed high levels of multidrug resistance. All isolates were resistant to cotrimoxazole, with over 90% showing resistance to penicillin (as inferred from oxacillin screening), and tetracycline. Conversely, chloramphenicol and vancomycin retained high effectiveness, suggesting that these remain viable treatment options in this context. These results are consistent with global trends in antimicrobial resistance but are particularly concerning in low-resource settings where treatment alternatives are limited. In contrast, Amritha et al. reported full susceptibility to penicillin, which may reflect differing prescribing practices or antibiotic stewardship policies between countries [14]. 

Several non-demographic factors were significantly associated with pneumococcal colonisation. Recent respiratory tract infections, and residence in northern Benin, part of the meningitis belt, were all positively correlated with increased carriage rates. These findings reinforce the notion that both individual-level and environmental factors contribute to pathogen circulation within healthcare environments [9]. While unvaccinated healthcare workers appeared to have higher pneumococcal colonisation rates, the odds ratio included 1, indicating that this association was not statistically significant despite a p-value below 0.05. This suggests that, although vaccination status may influence colonisation risk, the effect size in our study population was insufficient to demonstrate a clear association. 

Surprisingly, no significant association was observed between colonisation and cohabitation with children or the use of facemasks during patient care. This contrasts with the findings of Steurer et al., who identified living with young children as a key risk factor [12]. Despite the absence of a statistically significant association between mask use and pneumococcal colonisation in our study, this result should be interpreted with caution. Inconsistent adherence, suboptimal mask types, or overlapping infection control measures may have obscured a potential protective effect. Nevertheless, our findings are not consistent with the evidence supporting the role of masks in reducing transmission. For example, a previous study observed fewer positive respiratory cultures among neonates when staff used face masks, indirectly highlighting their preventive potential [17]. Although their study focused on neonatal outcomes, the implication remains relevant for healthcare worker carriage. As Altmann and colleagues acknowledged, their results were influenced by various contextual factors and call for replication in controlled, multi-centre studies. Similarly, our findings may reflect local limitations rather than a true absence of effect. Future studies should better isolate the role of masking through longitudinal, multicentric approaches. Building on the findings of asymptomatic carriage among paediatric healthcare workers, it is essential to highlight preventive measures that can mitigate these risks. Healthcare workers should adhere strictly to standard infection prevention and control practices. Key measures include proper hand hygiene following the WHO “Five Moments for Hand Hygiene,” consistent use of personal protective equipment such as masks when indicated, routine cleaning and disinfection of medical equipment and surfaces, and vaccination against relevant pathogens where available. Regular training and reinforcement of these practices can significantly reduce occupational exposure and help protect both healthcare personnel and vulnerable paediatric patients [18].

This study presents several strengths. It specifically targeted paediatric healthcare workers, a highly relevant population in the African meningitis belt. The use of paired oropharyngeal and nasopharyngeal swabs enhanced detection sensitivity, and combining culture with real-time PCR increased diagnostic accuracy. Moreover, the inclusion of antimicrobial resistance profiling and the assessment of multiple risk factors provided valuable insights for prevention strategies. However, some limitations should be noted. The cross-sectional design precludes causal inference. As PCR was performed only on nasopharyngeal samples, while oropharyngeal detection relied exclusively on culture, this methodological difference may have introduced differential misclassification between sites. Serotyping was not performed, and potential participation bias cannot be excluded.

## 5. Conclusions

This study highlights a substantial prevalence of asymptomatic colonisation by *Streptococcus pneumoniae* among healthcare workers in paediatric settings in Benin. These findings underscore the risk of horizontal transmission within healthcare facilities and highlight the importance of strengthening infection prevention measures, implementing targeted vaccination strategies, and integrating routine molecular surveillance to protect vulnerable paediatric populations.

## Figures and Tables

**Table 1:** Sociodemographic characteristics of the 261 paediatric healthcare workers who completed the questionnaire.

Variables (*n* = 261)	Frequency	Percentage (%)
**Age**		
18–24	76	29.1
25–34	97	37.2
35–44	55	21.1
≥45 years	33	12.6
**Sex**		
Male	102	39.1
Female	159	60.9
**Occupational Group**		
Paediatrician	11	4.2
Medical doctors	76	29.1
Nurses	127	48.7
Nursing assistants	47	18.0
**Clinical Ward**		
Neonatology	86	33.0
Paediatric	135	51.7
Paediatric emergency	40	15.3
**Department**		
Atacora	34	13.0
Borgou	86	33.0
Littoral	113	43.3
Mono	28	10.7

**Table 2:** Comparison of carriage detection by culture in nasopharyngeal and oropharyngeal swabs.

Sampling Site	No. of Samples	Positive *S. pneumoniae*, *n* (%)	Positive *H. influenzae*, *n* (%)
Oropharynx	261	124 (47.5%)	10 (3.8%)
Nasopharynx	261	13 (5%)	1 (0.38%)

**Table 3:** Sociodemographic characteristics associated with culture-confirmed pneumococcal colonisation among paediatric healthcare workers.

Sociodemographic Characteristics	*n* = 261 (%)	Colonised, *n* (%)	Non-Colonised, *n* (%)	OR [95%CI]	Chi-Square, *p* Value *
**Sex**				0.79 [0.4–1.2]	0.087
Male	102 (39.1)	68 (52.7)	91 (68.9)		
Female	159 (60.9)	61 (47.3)	41 (31.1)		
**Age Groups**				1.02 [0.6–1.4]	0.021 *
18–24	76 (29.1)	55 (42.6)	21 (15.9)		
25–34	97 (37.2)	56 (43.4)	41 (31.1)		
35–44	55 (21.1)	10 (7.8)	45 (34.1)		
≥45	33 (12.6)	8 (6.2)	25 (18.9)		
**Profession**				1.31 [0.8–1.7]	0.001 *
Paediatrician	11 (4.2)	3 (2.3)	8 (6.1)		
Medical doctor	76 (29.1)	59 (45.7)	17 (12.9)		
Nurse	127 (48.7)	48 (37.2)	79 (59.8)		
Nursing assistant	47 (18.0)	19 (14.7)	28 (21.2)		
**Work Setting**				0.79 [0.4–1.2]	0.161
Neonatology	86 (33.0)	32 (24.8)	54 (40.9)		
Paediatrics	135 (51.7)	86 (66.7)	49 (37.1)		
Paediatric emergencies	40 (15.3)	11 (8.5)	29 (22.0)		

* *p* values ˂ 0.05 considered statistically significant.

**Table 4:** Environmental and clinical determinants of *S. pneumoniae* carriage in healthcare workers.

Factors	*n* = 261 (%)	Colonised, *n* (%)	Non-Colonised, *n* (%)	OR [95%CI]	Chi-Square *p* Value *
**Cohabitation with children under 10 years**				0.79 [0.7–2.2]	0.091
Yes	121 (46.4)	76 (58.9)	64 (48.5)		
No	140 (53.6)	53 (41.1)	68 (51.5)		
**Recent respiratory tract infection**				1.3 [1.1–3.4]	0.022 *
Yes	58 (22.2)	21 (16.3)	37 (28.0)		
No	203 (77.8)	108 (83.7)	95 (72.0)		
**Vaccination status**				1.1 [0.4–1.6]	0.002 *
Yes	95 (36.4)	35 (27.1)	60 (45.5)		
No	166 (63.6)	94 (72.8)	72 (54.5)		
**Residence in the meningitis belt** **(northern Benin)**				1.9 [1.3–4.1]	0.001*
Yes	120 (46)	80 (62)	40 (33.3)		
No	141 (54)	49 (38)	92 (69.7)		
**Routine use of face masks**				0.61 [0.3–1.9]	0.694
Yes	84 (32.2)	43 (33.3)	41 (31.1)		
No	177 (67.8)	86 (66.7)	91 (68.9)		

* *p* values ˂ 0.05 considered statistically significant.

## Data Availability

The data supporting the findings of this study are available from the corresponding author upon reasonable request. Due to privacy and ethical restrictions, raw data are not publicly shared.

## Supplementary Materials

The following supporting information is available online at: https://www.mdpi.com/article/10.3390/tropicalmed10090263/s1, Supplementary File S1: Structured questionnaire used for data collection.

## Abbreviations

The following abbreviations are used in this manuscript:

HCP	Healthcare professional
PCR	Polymerase chain reaction
*N. meningitidis*	*Neisseria meningitidis*
*H. influenzae*	*Haemophilus influenzae*
*S. pneumoniae*	*Streptococcus pneumoniae*
RT-PCR	Real-time polymerase chain reaction
LytA	Autolysin gene specific to *Streptococcus pneumoniae*
SodC	Superoxide dismutase gene specific to *Neisseria meningitidis*
Hpd3	Protein D gene specific to *Haemophilus influenzae*
CA-SFM	Antibiogram Committee of the French Society for Microbiology
