# Supplementary material for: Asymptomatic Pneumococcal Carriage, Antimicrobial Resistance, and Associated Risk Factors Among Paediatric Healthcare Workers in Benin
Source: Trop Med Infect Dis. 2025 Sep 15;10(9):263. doi: 10.3390/tropicalmed10090263 (PMC12474293; doi:10.3390/tropicalmed10090263)
Supplement: Supplementary file 1 [file tropicalmed-10-00263-s001.zip › tropicalmed-3792776-SI.pdf]

RESEARCH TITLE: Epidemiological profile and characterisation of meningitis-associated pathogenic bacteria isolated from healthcare workers in paediatric units.

Principal investigator: Chakir Ishola BELLO

Study Period: September 2023 – December 2024

Confidential – For research purposes only

QUESTIONNAIRE

Date ………/………./202 **Participant ID Code :**…………………….……….……..………………….

A 1. Department :……………………………..……… **A 2.** Hospital : ……………......…………………………

Q 1. Sex : Male /___/ Female /___/ **Q 2.** Age : /_____/ years

Q 3. Occupational group /____/ : **1**= Specialist ; **2**= General practitioner ; **3**= Nurse ; **4**= Nursing assistant

Q 4. Work unit /___/ : **1**= Neonatology ; **2** = Paediatrics ; **3** = Paediatric emergency

Q 5. Have you experienced respiratory tract infection symptoms in the past two weeks? /___/

1= Yes ; **2**= No

Q 6. Do you live with any children under 10 years of age? /___/ : **1**= Yes ; **2**= No

Q 7. Have you been vaccinated against meningitis? /____/ : **1**= Yes ; **2**= I don't know ; **3**= No

If **yes,** specify:

Q 8. Do you routinely wear a face mask during patient care? /___/ : **1**= Yes ; **2**= No
